# Ethyl {6-[6-(ethoxy­carbon­yl)picolin­amido­carbon­yl]picolinamido­carbon­yl}picolinate

**DOI:** 10.1107/S1600536808040932

**Published:** 2008-12-10

**Authors:** Xiao Li, Yaobing Wang, Chuanlang Zhan, Jiannian Yao

**Affiliations:** a Beijing National Laboratory for Molecular Sciences (BNLMS), Laboratory of Photochemistry, Institute of Chemistry, Chinese Academy of Sciences, Beijing 100190, People’s Republic of China, and Graduate University of the Chinese Academy of Sciences (GUCAS), Beijing 100049, People’s Republic of China

## Abstract

The title mol­ecule, C_25_H_21_N_5_O_8_, adopts a helical conformation, which is stabilized by two intra­molecular bifurcated N—H⋯(N,N) hydrogen bonds.

## Related literature

For a review on aromatic oligoamides (AOAs), see, for example: Huc (2004 ▶). For related compounds, see: Li *et al.* (2008 ▶).
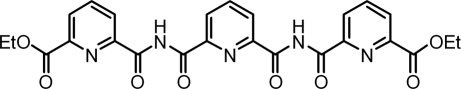


## Experimental

### 

#### Crystal data

C_25_H_21_N_5_O_8_
*M* *_r_* = 519.47
Monoclinic, 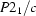

*a* = 7.4952 (8) Å
*b* = 19.998 (2) Å
*c* = 15.9966 (17) Å
β = 96.376 (1)°
*V* = 2382.8 (4) Å^3^
*Z* = 4
Mo *K*α radiation
μ = 0.11 mm^−1^
*T* = 113 (2) K
0.32 × 0.22 × 0.18 mm

#### Data collection

Rigaku Saturn diffractometer
Absorption correction: multi-scan (CrystalcClear; Rigaku, 1999 ▶) *T*_min_ = 0.966, *T*_max_ = 0.980
30537 measured reflections
6101 independent reflections
5457 reflections with *I* > 2σ(*I*)
*R*_int_ = 0.025

#### Refinement

*R*[*F* ^2^ > 2σ(*F* ^2^)] = 0.037
*wR*(*F* ^2^) = 0.096
*S* = 1.08
6101 reflections
354 parameters
H atoms treated by a mixture of independent and constrained refinement
Δρ_max_ = 0.32 e Å^−3^
Δρ_min_ = −0.17 e Å^−3^

### 

Data collection: *CrystalClear* (Rigaku, 1999 ▶); cell refinement: *CrystalClear*; data reduction: *CrystalClear*; program(s) used to solve structure: *SHELXS97* (Sheldrick, 2008 ▶); program(s) used to refine structure: *SHELXL97* (Sheldrick, 2008 ▶); molecular graphics: *SHELXTL* (Sheldrick, 2008 ▶); software used to prepare material for publication: *CrystalStructure* (Rigaku, 1999 ▶).

## Supplementary Material

Crystal structure: contains datablocks I, global. DOI: 10.1107/S1600536808040932/hb2864sup1.cif

Structure factors: contains datablocks I. DOI: 10.1107/S1600536808040932/hb2864Isup2.hkl

Additional supplementary materials:  crystallographic information; 3D view; checkCIF report

## Figures and Tables

**Table 1:** Hydrogen-bond geometry (Å, °)

*D*—H⋯*A*	*D*—H	H⋯*A*	*D*⋯*A*	*D*—H⋯*A*
N4—H4⋯N3	0.885 (14)	2.153 (14)	2.6131 (13)	111.7 (11)
N4—H4⋯N5	0.885 (14)	2.158 (14)	2.6297 (12)	112.8 (11)
N2—H2⋯N3	0.878 (15)	2.148 (14)	2.6006 (12)	111.4 (11)
N2—H2⋯N1	0.878 (15)	2.151 (14)	2.6329 (12)	114.0 (11)

**Fig. 1.**
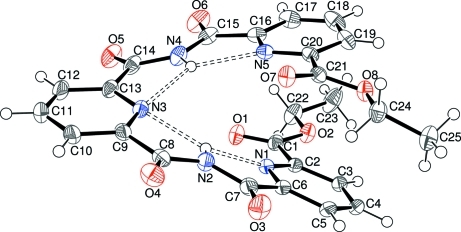
The molecular structure of (I) showing 50% displacement ellipsoids for the non-hydrogen atoms. The hydrogen bonds are shown as double-dashed lines.

**Fig. 2.**
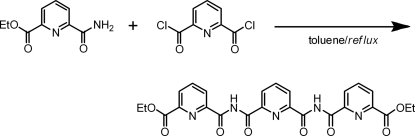
The formation of the title compound.

## supplementary crystallographic information

### Comment

The stucture of the title compound is shown in Fig. 1. Dimensions are available in the archived CIF. The hydrogen bonds are listed in Table 1. For background, see for example: Huc (2004). For related compounds, see: Li *et al.* (2008).

### Experimental

The title compound was obtained from 2-ethoxycarbonyl- 6-pyridinoyl amide and 2,6-pyridinoyl dichloride and recrystallised from DMF/ethyl ether to yield colourless prisms of (I).

### Refinement

The N-bound hydrogen atoms were located in a difference map and freely refined. The C-bound hydrogen atoms were geometrically placed (C—H = 0.95–0.99Å) and refined as riding with U_iso_(H) = 1.2U_eq_(C) or 1.5U_eq_(methyl C).

### Crystal data

**Table:**

C_25_H_21_N_5_O_8_	*F*(000) = 1080
*M**_r_* = 519.47	*D*_x_ = 1.448 Mg m^−^^3^
Monoclinic, *P*2_1_/*c*	Mo *K*α radiation, λ = 0.71070 Å
Hall symbol: -P 2ybc	Cell parameters from 6439 reflections
*a* = 7.4952 (8) Å	θ = 2.6–26.0°
*b* = 19.998 (2) Å	µ = 0.11 mm^−^^1^
*c* = 15.9966 (17) Å	*T* = 113 K
β = 96.376 (1)°	Prism, colorless
*V* = 2382.8 (4) Å^3^	0.32 × 0.22 × 0.18 mm
*Z* = 4	

### Data collection

**Table:**

Rigaku Saturn diffractometer	6101 independent reflections
Radiation source: rotating anode	5457 reflections with *I* > 2σ(*I*)
confocal	*R*_int_ = 0.025
Detector resolution: 7.31 pixels mm^-1^	θ_max_ = 28.7°, θ_min_ = 2.4°
ω scans	*h* = −10→10
Absorption correction: multi-scan (CrystalcClear; Rigaku, 1999)	*k* = −27→24
*T*_min_ = 0.966, *T*_max_ = 0.980	*l* = −21→21
30537 measured reflections	

### Refinement

**Table:**

Refinement on *F*^2^	Primary atom site location: structure-invariant direct methods
Least-squares matrix: full	Secondary atom site location: difference Fourier map
*R*[*F*^2^ > 2σ(*F*^2^)] = 0.037	Hydrogen site location: inferred from neighbouring sites
*wR*(*F*^2^) = 0.096	H atoms treated by a mixture of independent and constrained refinement
*S* = 1.08	*w* = 1/[σ^2^(*F*_o_^2^) + (0.0481*P*)^2^ + 0.4996*P*] where *P* = (*F*_o_^2^ + 2*F*_c_^2^)/3
6101 reflections	(Δ/σ)_max_ = 0.001
354 parameters	Δρ_max_ = 0.32 e Å^−^^3^
0 restraints	Δρ_min_ = −0.17 e Å^−^^3^

### Special details

**Table:**

Geometry. All e.s.d.'s (except the e.s.d. in the dihedral angle between two l.s. planes) are estimated using the full covariance matrix. The cell e.s.d.'s are taken into account individually in the estimation of e.s.d.'s in distances, angles and torsion angles; correlations between e.s.d.'s in cell parameters are only used when they are defined by crystal symmetry. An approximate (isotropic) treatment of cell e.s.d.'s is used for estimating e.s.d.'s involving l.s. planes.
Refinement. Refinement of *F*^2^ against ALL reflections. The weighted *R*-factor *wR* and goodness of fit *S* are based on *F*^2^, conventional *R*-factors *R* are based on *F*, with *F* set to zero for negative *F*^2^. The threshold expression of *F*^2^ > σ(*F*^2^) is used only for calculating *R*-factors(gt) *etc*. and is not relevant to the choice of reflections for refinement. *R*-factors based on *F*^2^ are statistically about twice as large as those based on *F*, and *R*- factors based on ALL data will be even larger.

### Fractional atomic coordinates and isotropic or equivalent isotropic displacement parameters (Å^2^)

**Table:**

	*x*	*y*	*z*	*U*_iso_*/*U*_eq_	
O1	−0.13320 (11)	0.87694 (4)	0.72665 (5)	0.02977 (17)	
O2	0.06124 (11)	0.85871 (4)	0.63149 (5)	0.02910 (17)	
O3	−0.16781 (12)	0.59423 (4)	0.88294 (6)	0.0395 (2)	
O4	−0.33384 (11)	0.65393 (4)	1.02334 (5)	0.03521 (19)	
O5	−0.24510 (13)	0.99672 (4)	0.94666 (5)	0.0397 (2)	
O6	0.08420 (13)	1.00415 (4)	0.86648 (5)	0.0391 (2)	
O7	0.21781 (10)	0.70449 (4)	0.93765 (4)	0.02615 (16)	
O8	0.37042 (9)	0.67410 (4)	0.82982 (4)	0.02587 (16)	
N1	−0.10247 (10)	0.74283 (4)	0.76977 (5)	0.02135 (16)	
N2	−0.21026 (12)	0.70511 (4)	0.91366 (5)	0.02446 (18)	
N3	−0.25843 (11)	0.82284 (4)	0.97648 (5)	0.02253 (17)	
N4	−0.04508 (12)	0.91296 (4)	0.92339 (5)	0.02641 (18)	
N5	0.20551 (11)	0.83332 (4)	0.87730 (5)	0.02099 (16)	
C1	−0.04508 (13)	0.83975 (5)	0.68831 (6)	0.02336 (19)	
C2	−0.03851 (12)	0.76501 (5)	0.70001 (6)	0.02167 (19)	
C3	0.02896 (13)	0.72245 (5)	0.64172 (6)	0.0245 (2)	
H3	0.0764	0.7400	0.5936	0.029*	
C4	0.02507 (13)	0.65399 (5)	0.65576 (6)	0.0264 (2)	
H4A	0.0657	0.6237	0.6162	0.032*	
C5	−0.03894 (13)	0.63047 (5)	0.72829 (7)	0.0262 (2)	
H5	−0.0419	0.5839	0.7398	0.031*	
C6	−0.09874 (12)	0.67674 (5)	0.78370 (6)	0.02280 (19)	
C7	−0.16141 (13)	0.65290 (5)	0.86502 (7)	0.0260 (2)	
C8	−0.29462 (13)	0.70359 (5)	0.98611 (6)	0.0248 (2)	
C9	−0.34013 (13)	0.77315 (5)	1.01301 (6)	0.0241 (2)	
C10	−0.46393 (14)	0.78407 (7)	1.07012 (7)	0.0334 (2)	
H10	−0.5180	0.7478	1.0961	0.040*	
C11	−0.50571 (15)	0.84953 (7)	1.08782 (8)	0.0400 (3)	
H11	−0.5904	0.8588	1.1263	0.048*	
C12	−0.42408 (15)	0.90164 (6)	1.04950 (7)	0.0353 (3)	
H12	−0.4530	0.9468	1.0603	0.042*	
C13	−0.29831 (14)	0.88586 (5)	0.99470 (6)	0.0263 (2)	
C14	−0.19693 (15)	0.93888 (5)	0.95273 (6)	0.0287 (2)	
C15	0.08115 (15)	0.94475 (5)	0.88163 (6)	0.0272 (2)	
C16	0.22015 (13)	0.89727 (5)	0.85479 (6)	0.0240 (2)	
C17	0.35075 (15)	0.92050 (6)	0.80694 (7)	0.0318 (2)	
H17	0.3587	0.9667	0.7939	0.038*	
C18	0.46885 (15)	0.87489 (6)	0.77877 (8)	0.0350 (3)	
H18	0.5603	0.8892	0.7462	0.042*	
C19	0.45178 (14)	0.80778 (6)	0.79891 (7)	0.0290 (2)	
H19	0.5286	0.7751	0.7788	0.035*	
C20	0.31961 (12)	0.78940 (5)	0.84913 (6)	0.02153 (19)	
C21	0.29545 (12)	0.71868 (5)	0.87815 (6)	0.02146 (19)	
C22	0.06263 (18)	0.92999 (5)	0.61175 (7)	0.0342 (2)	
H22A	0.0827	0.9568	0.6640	0.041*	
H22B	−0.0532	0.9435	0.5806	0.041*	
C23	0.2126 (2)	0.94046 (6)	0.55884 (9)	0.0482 (4)	
H23A	0.3267	0.9282	0.5912	0.072*	
H23B	0.2163	0.9876	0.5423	0.072*	
H23C	0.1931	0.9124	0.5084	0.072*	
C24	0.35652 (15)	0.60428 (5)	0.85650 (7)	0.0275 (2)	
H24A	0.2315	0.5941	0.8666	0.033*	
H24B	0.4356	0.5965	0.9094	0.033*	
C25	0.41238 (17)	0.56010 (6)	0.78771 (7)	0.0352 (2)	
H25A	0.3290	0.5662	0.7366	0.053*	
H25B	0.4104	0.5133	0.8056	0.053*	
H25C	0.5341	0.5721	0.7762	0.053*	
H4	−0.0313 (18)	0.8692 (7)	0.9284 (8)	0.036 (4)*	
H2	−0.1908 (19)	0.7449 (8)	0.8932 (9)	0.040 (4)*	

### Atomic displacement parameters (Å^2^)

**Table:**

	*U*^11^	*U*^22^	*U*^33^	*U*^12^	*U*^13^	*U*^23^
O1	0.0356 (4)	0.0277 (4)	0.0269 (4)	0.0077 (3)	0.0077 (3)	−0.0011 (3)
O2	0.0378 (4)	0.0242 (4)	0.0268 (4)	0.0026 (3)	0.0104 (3)	0.0007 (3)
O3	0.0507 (5)	0.0201 (4)	0.0503 (5)	−0.0036 (3)	0.0176 (4)	0.0003 (3)
O4	0.0350 (4)	0.0360 (4)	0.0355 (4)	−0.0062 (3)	0.0075 (3)	0.0099 (3)
O5	0.0549 (5)	0.0247 (4)	0.0386 (5)	0.0185 (4)	0.0005 (4)	−0.0020 (3)
O6	0.0650 (6)	0.0165 (4)	0.0358 (4)	−0.0003 (4)	0.0059 (4)	0.0058 (3)
O7	0.0308 (4)	0.0222 (4)	0.0266 (4)	0.0031 (3)	0.0085 (3)	0.0035 (3)
O8	0.0267 (4)	0.0239 (4)	0.0280 (4)	0.0045 (3)	0.0076 (3)	0.0009 (3)
N1	0.0193 (4)	0.0215 (4)	0.0231 (4)	−0.0004 (3)	0.0013 (3)	−0.0035 (3)
N2	0.0272 (4)	0.0203 (4)	0.0269 (4)	−0.0018 (3)	0.0075 (3)	0.0004 (3)
N3	0.0231 (4)	0.0256 (4)	0.0186 (4)	0.0055 (3)	0.0007 (3)	−0.0008 (3)
N4	0.0374 (5)	0.0147 (4)	0.0277 (4)	0.0050 (3)	0.0059 (3)	0.0009 (3)
N5	0.0218 (4)	0.0188 (4)	0.0218 (4)	−0.0021 (3)	−0.0004 (3)	0.0022 (3)
C1	0.0249 (5)	0.0267 (5)	0.0179 (4)	0.0022 (4)	0.0001 (3)	−0.0014 (3)
C2	0.0187 (4)	0.0245 (5)	0.0211 (4)	0.0011 (3)	−0.0011 (3)	−0.0036 (3)
C3	0.0209 (4)	0.0301 (5)	0.0218 (4)	0.0016 (4)	0.0001 (3)	−0.0058 (4)
C4	0.0223 (4)	0.0286 (5)	0.0278 (5)	0.0021 (4)	−0.0002 (4)	−0.0109 (4)
C5	0.0234 (5)	0.0220 (5)	0.0325 (5)	−0.0012 (4)	0.0005 (4)	−0.0084 (4)
C6	0.0194 (4)	0.0218 (5)	0.0269 (5)	−0.0022 (3)	0.0013 (3)	−0.0053 (4)
C7	0.0241 (5)	0.0208 (5)	0.0334 (5)	−0.0028 (4)	0.0052 (4)	−0.0024 (4)
C8	0.0197 (4)	0.0309 (5)	0.0239 (5)	−0.0021 (4)	0.0020 (3)	0.0026 (4)
C9	0.0190 (4)	0.0340 (5)	0.0189 (4)	0.0008 (4)	0.0009 (3)	−0.0006 (4)
C10	0.0233 (5)	0.0522 (7)	0.0254 (5)	−0.0044 (5)	0.0061 (4)	−0.0067 (5)
C11	0.0255 (5)	0.0603 (8)	0.0357 (6)	0.0018 (5)	0.0099 (4)	−0.0177 (6)
C12	0.0280 (5)	0.0441 (7)	0.0332 (6)	0.0112 (5)	0.0014 (4)	−0.0141 (5)
C13	0.0260 (5)	0.0299 (5)	0.0218 (4)	0.0099 (4)	−0.0017 (4)	−0.0048 (4)
C14	0.0375 (6)	0.0245 (5)	0.0231 (5)	0.0103 (4)	−0.0013 (4)	−0.0029 (4)
C15	0.0412 (6)	0.0179 (4)	0.0219 (4)	−0.0010 (4)	0.0004 (4)	0.0022 (3)
C16	0.0281 (5)	0.0200 (5)	0.0230 (4)	−0.0040 (4)	−0.0017 (4)	0.0036 (3)
C17	0.0330 (5)	0.0279 (5)	0.0338 (5)	−0.0085 (4)	0.0005 (4)	0.0108 (4)
C18	0.0250 (5)	0.0423 (7)	0.0383 (6)	−0.0051 (4)	0.0060 (4)	0.0166 (5)
C19	0.0203 (4)	0.0365 (6)	0.0304 (5)	0.0028 (4)	0.0040 (4)	0.0096 (4)
C20	0.0183 (4)	0.0236 (5)	0.0220 (4)	0.0000 (3)	−0.0007 (3)	0.0037 (3)
C21	0.0180 (4)	0.0229 (5)	0.0229 (4)	0.0024 (3)	0.0001 (3)	0.0015 (3)
C22	0.0510 (7)	0.0238 (5)	0.0287 (5)	0.0041 (5)	0.0082 (5)	0.0031 (4)
C23	0.0808 (10)	0.0273 (6)	0.0419 (7)	−0.0084 (6)	0.0309 (7)	−0.0045 (5)
C24	0.0320 (5)	0.0221 (5)	0.0288 (5)	0.0063 (4)	0.0056 (4)	0.0017 (4)
C25	0.0420 (6)	0.0298 (6)	0.0350 (6)	0.0093 (5)	0.0095 (5)	−0.0033 (4)

### Geometric parameters (Å, °)

**Table:**

O1—C1	1.2064 (12)	C8—C9	1.5063 (15)
O2—C1	1.3289 (12)	C9—C10	1.3898 (14)
O2—C22	1.4603 (13)	C10—C11	1.3825 (18)
O3—C7	1.2100 (13)	C10—H10	0.9500
O4—C8	1.2109 (13)	C11—C12	1.3854 (19)
O5—C14	1.2123 (13)	C11—H11	0.9500
O6—C15	1.2131 (13)	C12—C13	1.3930 (15)
O7—C21	1.2034 (12)	C12—H12	0.9500
O8—C21	1.3434 (12)	C13—C14	1.5052 (16)
O8—C24	1.4671 (12)	C15—C16	1.5068 (15)
N1—C2	1.3382 (13)	C16—C17	1.3880 (15)
N1—C6	1.3401 (13)	C17—C18	1.3809 (17)
N2—C7	1.3760 (13)	C17—H17	0.9500
N2—C8	1.3809 (13)	C18—C19	1.3893 (16)
N2—H2	0.878 (15)	C18—H18	0.9500
N3—C13	1.3348 (13)	C19—C20	1.3924 (14)
N3—C9	1.3354 (13)	C19—H19	0.9500
N4—C15	1.3732 (14)	C20—C21	1.5057 (13)
N4—C14	1.3796 (14)	C22—C23	1.4949 (18)
N4—H4	0.885 (14)	C22—H22A	0.9900
N5—C16	1.3364 (12)	C22—H22B	0.9900
N5—C20	1.3384 (13)	C23—H23A	0.9800
C1—C2	1.5064 (14)	C23—H23B	0.9800
C2—C3	1.3979 (13)	C23—H23C	0.9800
C3—C4	1.3882 (15)	C24—C25	1.5064 (14)
C3—H3	0.9500	C24—H24A	0.9900
C4—C5	1.3859 (15)	C24—H24B	0.9900
C4—H4A	0.9500	C25—H25A	0.9800
C5—C6	1.3895 (14)	C25—H25B	0.9800
C5—H5	0.9500	C25—H25C	0.9800
C6—C7	1.5086 (14)		
			
C1—O2—C22	116.47 (8)	C12—C13—C14	122.10 (10)
C21—O8—C24	114.60 (8)	O5—C14—N4	125.50 (11)
C2—N1—C6	117.56 (8)	O5—C14—C13	123.18 (10)
C7—N2—C8	129.30 (9)	N4—C14—C13	111.32 (8)
C7—N2—H2	114.2 (9)	O6—C15—N4	125.43 (11)
C8—N2—H2	116.3 (9)	O6—C15—C16	122.08 (10)
C13—N3—C9	118.83 (9)	N4—C15—C16	112.49 (8)
C15—N4—C14	129.10 (9)	N5—C16—C17	123.57 (10)
C15—N4—H4	115.0 (9)	N5—C16—C15	116.50 (9)
C14—N4—H4	115.7 (9)	C17—C16—C15	119.90 (9)
C16—N5—C20	117.56 (9)	C18—C17—C16	118.41 (10)
O1—C1—O2	125.14 (10)	C18—C17—H17	120.8
O1—C1—C2	124.11 (9)	C16—C17—H17	120.8
O2—C1—C2	110.75 (8)	C17—C18—C19	118.91 (10)
N1—C2—C3	122.96 (9)	C17—C18—H18	120.5
N1—C2—C1	114.99 (8)	C19—C18—H18	120.5
C3—C2—C1	122.04 (9)	C18—C19—C20	118.59 (10)
C4—C3—C2	118.48 (10)	C18—C19—H19	120.7
C4—C3—H3	120.8	C20—C19—H19	120.7
C2—C3—H3	120.8	N5—C20—C19	122.90 (9)
C5—C4—C3	119.04 (9)	N5—C20—C21	114.16 (8)
C5—C4—H4A	120.5	C19—C20—C21	122.92 (9)
C3—C4—H4A	120.5	O7—C21—O8	124.66 (9)
C4—C5—C6	118.31 (10)	O7—C21—C20	123.41 (9)
C4—C5—H5	120.8	O8—C21—C20	111.94 (8)
C6—C5—H5	120.8	O2—C22—C23	106.44 (9)
N1—C6—C5	123.57 (9)	O2—C22—H22A	110.4
N1—C6—C7	116.89 (8)	C23—C22—H22A	110.4
C5—C6—C7	119.53 (9)	O2—C22—H22B	110.4
O3—C7—N2	125.58 (10)	C23—C22—H22B	110.4
O3—C7—C6	122.35 (9)	H22A—C22—H22B	108.6
N2—C7—C6	112.06 (8)	C22—C23—H23A	109.5
O4—C8—N2	126.13 (10)	C22—C23—H23B	109.5
O4—C8—C9	122.81 (9)	H23A—C23—H23B	109.5
N2—C8—C9	111.02 (8)	C22—C23—H23C	109.5
N3—C9—C10	122.87 (10)	H23A—C23—H23C	109.5
N3—C9—C8	115.64 (8)	H23B—C23—H23C	109.5
C10—C9—C8	121.45 (10)	O8—C24—C25	108.13 (8)
C11—C10—C9	117.80 (11)	O8—C24—H24A	110.1
C11—C10—H10	121.1	C25—C24—H24A	110.1
C9—C10—H10	121.1	O8—C24—H24B	110.1
C10—C11—C12	120.02 (10)	C25—C24—H24B	110.1
C10—C11—H11	120.0	H24A—C24—H24B	108.4
C12—C11—H11	120.0	C24—C25—H25A	109.5
C11—C12—C13	118.10 (11)	C24—C25—H25B	109.5
C11—C12—H12	121.0	H25A—C25—H25B	109.5
C13—C12—H12	121.0	C24—C25—H25C	109.5
N3—C13—C12	122.34 (11)	H25A—C25—H25C	109.5
N3—C13—C14	115.55 (9)	H25B—C25—H25C	109.5
			
C22—O2—C1—O1	−3.25 (15)	C9—N3—C13—C12	−0.98 (14)
C22—O2—C1—C2	177.85 (8)	C9—N3—C13—C14	178.44 (8)
C6—N1—C2—C3	0.73 (13)	C11—C12—C13—N3	1.89 (16)
C6—N1—C2—C1	−179.54 (8)	C11—C12—C13—C14	−177.48 (10)
O1—C1—C2—N1	−15.36 (14)	C15—N4—C14—O5	−0.58 (18)
O2—C1—C2—N1	163.55 (8)	C15—N4—C14—C13	179.72 (9)
O1—C1—C2—C3	164.37 (10)	N3—C13—C14—O5	162.22 (10)
O2—C1—C2—C3	−16.71 (12)	C12—C13—C14—O5	−18.36 (16)
N1—C2—C3—C4	1.73 (14)	N3—C13—C14—N4	−18.07 (12)
C1—C2—C3—C4	−177.98 (8)	C12—C13—C14—N4	161.35 (9)
C2—C3—C4—C5	−2.40 (14)	C14—N4—C15—O6	3.77 (18)
C3—C4—C5—C6	0.72 (14)	C14—N4—C15—C16	−175.83 (9)
C2—N1—C6—C5	−2.58 (14)	C20—N5—C16—C17	−2.57 (14)
C2—N1—C6—C7	176.59 (8)	C20—N5—C16—C15	175.38 (8)
C4—C5—C6—N1	1.87 (15)	O6—C15—C16—N5	178.10 (10)
C4—C5—C6—C7	−177.27 (9)	N4—C15—C16—N5	−2.29 (12)
C8—N2—C7—O3	−8.40 (18)	O6—C15—C16—C17	−3.88 (15)
C8—N2—C7—C6	170.93 (9)	N4—C15—C16—C17	175.74 (9)
N1—C6—C7—O3	178.12 (10)	N5—C16—C17—C18	2.05 (16)
C5—C6—C7—O3	−2.68 (15)	C15—C16—C17—C18	−175.83 (10)
N1—C6—C7—N2	−1.24 (12)	C16—C17—C18—C19	0.37 (16)
C5—C6—C7—N2	177.96 (9)	C17—C18—C19—C20	−2.07 (16)
C7—N2—C8—O4	4.13 (18)	C16—N5—C20—C19	0.70 (14)
C7—N2—C8—C9	−173.76 (9)	C16—N5—C20—C21	179.34 (8)
C13—N3—C9—C10	−0.73 (14)	C18—C19—C20—N5	1.58 (15)
C13—N3—C9—C8	177.19 (8)	C18—C19—C20—C21	−176.94 (9)
O4—C8—C9—N3	167.30 (9)	C24—O8—C21—O7	−1.92 (13)
N2—C8—C9—N3	−14.72 (12)	C24—O8—C21—C20	178.12 (8)
O4—C8—C9—C10	−14.75 (15)	N5—C20—C21—O7	−19.00 (13)
N2—C8—C9—C10	163.23 (9)	C19—C20—C21—O7	159.64 (10)
N3—C9—C10—C11	1.43 (15)	N5—C20—C21—O8	160.96 (8)
C8—C9—C10—C11	−176.37 (10)	C19—C20—C21—O8	−20.40 (13)
C9—C10—C11—C12	−0.44 (17)	C1—O2—C22—C23	170.48 (10)
C10—C11—C12—C13	−1.12 (17)	C21—O8—C24—C25	168.94 (8)

### Hydrogen-bond geometry (Å, °)

**Table:**

*D*—H···*A*	*D*—H	H···*A*	*D*···*A*	*D*—H···*A*
N4—H4···N3	0.885 (14)	2.153 (14)	2.6131 (13)	111.7 (11)
N4—H4···N5	0.885 (14)	2.158 (14)	2.6297 (12)	112.8 (11)
N2—H2···N3	0.878 (15)	2.148 (14)	2.6006 (12)	111.4 (11)
N2—H2···N1	0.878 (15)	2.151 (14)	2.6329 (12)	114.0 (11)
